# Startling speech: eliciting prepared speech using startling auditory stimulus

**DOI:** 10.3389/fpsyg.2014.01082

**Published:** 2014-09-26

**Authors:** Chenhao Chiu, Bryan Gick

**Affiliations:** ^1^Department of Linguistics, University of British ColumbiaVancouver, BC, Canada; ^2^Haskins LaboratoriesNew Haven, CT, USA

**Keywords:** speech planning, feedforward control, speech motor control, startling auditory stimulus, the StartReact effect

Speech research has recently seen a good deal of activity surrounding forward models (Tian and Poeppel, 2012; Pickering and Garrod, 2013; Scott, 2013), expanding on a long tradition of work in preprogramming of speech motor plans (e.g., Lashley, 1951; Keele, 1981; Klapp, 2003). Despite the volume of activity and interest in this area, few studies have offered insight into the detailed content of these forward plans. The content of such plans should presumably specify, at minimum, those aspects of speech that are essential in determining linguistic contrast, independent of the many aspects of a physical speech utterance that may be determined or altered through feedback mechanisms. Our previous work has attempted to uncover some of the detailed content of such forward plans using behavioral methods (Scott et al., 2013), while other studies have used neuroimaging methods (e.g., Heinks-Maldonado et al., 2006). Both approaches have given suggestive results, though not without concerns regarding interpretation (Niziolek et al., 2013).

A novel experimental methodology employing startling auditory stimuli (SAS, >120 dB) has been used to demonstrate the execution of prepared non-speech motor behaviors (e.g., head rotation and upper limb movements) with little or no interference from feedback regulation (Valls-Solé et al., 1999; Oude Nijhuis et al., 2007; Carlsen et al., 2012). Accelerated release of prepared movements (as short as 70 ms for EMG response onset) in response to SAS has been termed the StartReact effect (Valls-Solé et al., 1999, 2008). Because of their very short onset latency, SAS-induced actions may be fully executed before they are affected by sensory feedback, thus enabling study of the forward plan. It is our opinion that this experimental paradigm is ideally suited for investigating speech production and uncovering the detailed contents of forward speech plans.

Early analyses hypothesized that the rapid release of SAS-induced responses is the result of triggering subcortically stored information with faster neural transmissions (Carlsen et al., 2004; see also Castellote et al., 2012; Nonnekes et al., 2014). However, recent transcranial magnetic stimulation (TMS) studies show that the StartReact effect may not be limited to subcortically stored programs, but can also be observed in cortically dependent processes (Alibiglou and MacKinnon, 2012; Stevenson et al., 2014). These studies found that, when a cortical silent period was induced by applying TMS to motor cortex, the StartReact response was delayed in startle trials. If only subcortical processes were involved, TMS should not have affected the StartReact response. The delay in the StartReact response suggests that the pathways for a StartReact response would be mediated by, rather than bypassing, cortical areas.

Following these studies of finger movement, Stevenson et al. (2014) apply the startle paradigm to prepared spoken syllables, observing that voluntary lip movements were released at shorter latencies by a SAS, while the timing of kinematic displacement remained unaffected and formant profiles were performed as intended with no disruption. These results support the view that prepared syllables encode sufficient kinematic and acoustic information as part of the forward plan, and that this information may be subject to rapid release by a SAS. Extending this paradigm to pitch control in speech, Chiu and Gick (in press) show that a SAS induces an elevated pitch level in prepared syllables. Speakers show no evidence of an attempt to correct this elevated pitch to a baseline level even though auditory and somatosensory feedback is likely available before the end of the response. These findings raise questions as to the extent to which feedback information may affect SAS-induced responses, and suggest that uncorrected contents of forward speech plans may be observable even for longer (i.e., multisyllabic) responses using SAS.

The observed StartReact effect in syllable production also suggests that SAS-induced motor tasks, including upper limb, and speech movements, may involve similar neural pathways. It is noteworthy that the StartReact pathways involve similar pathways for speech production. As summarized in Carlsen et al. (2012), the StartReact response is mediated via an ascending thalamo-cortical pathway, generated by activation from reticular formation exerting on thalamus. Increased activation in thalamus provides inputs to primary motor cortex to initiate the cortically prepared movement via a descending corticospinal pathway. Similarly, speech production may also rely on thalamo-cortical circuits, and a descending corticospinal pathway. Specifically, receiving inputs from cerebellum, thalamus projects to primary motor cortex and Broca's area, and the commands are mediated via putamen and reticular formation and sent down to the phonatory motoneurones in the spine (Iwata et al., 1996; Jürgens, 2002; Guenther et al., 2006). Given that speech production involves a similar thalamo-cortical pathway to the one found in upper limb movements, upper limb movements, and speech movements may share the same StartReact pathways when elicited by a SAS. Shorter reaction times in StartReact responses are accounted for by increased neuron activation reaching faster above initiation threshold (see Carlsen et al., 2012 for details).

Similar to the calculation of the required time span for voluntary limbic movements, we can also conservatively calculate the time required for a speech response. First, Schroeder and Foxe (2002) report a response latency of 10 ~ 25 ms from the onset of auditory stimulus to the activation in the auditory cortex. Second, another 5 ~ 10 ms is required for the stimulus to be conducted between the lateral lemniscus and the thalamus for auditorily-evoked responses (Stockard et al., 1977). Third, transcortical and thalamus-primary motor cortex transmissions require 2 ~ 4 ms for conduction (Guenther et al., 2006; Carlsen et al., 2012). Last, the orofacial muscle EMG response to TMS on the face area of the motor cortex has a latency of about 11 ~ 12 ms (Meyer et al., 1994) and the motor time for the muscle movement is delayed by 30 ms. Adding these values gives a minimum of 58 ~ 81 ms lag time in response to a SAS. As reported in Stevenson et al. (2014), the onset of SAS-induced responses is 75 ms, suggesting that the shared neural pathway used for limb movements and speech movements does lead to a StartReact effect for speech movement.

Insofar as programming is necessary for speech production, we believe that the SAS methodology provides a new perspective that can help us to uncover the kinematic and linguistic contents of forward speech plans. Neural correlates and pathways for SAS-induced responses also support the view that SAS-induced speech responses may contain unaltered details of speech plans, allowing researchers a window into forward speech planning that bypasses afferent feedback information.

## Conflict of interest statement

The authors declare that the research was conducted in the absence of any commercial or financial relationships that could be construed as a potential conflict of interest.
